# Diagnosis of Hepatic Angiomyolipoma by Combination of Baseline and Contrast-Enhanced Ultrasound—A Prospective Study in Non-Cirrhotic Patients

**DOI:** 10.1371/journal.pone.0132290

**Published:** 2015-07-06

**Authors:** Rui Li, Chun-Lin Tang, Yi Zhang, Kuan-sheng Ma, Xiao-Hang Zhang, De-Yu Guo, Xiao-Chu Yan

**Affiliations:** 1 Dept. Hepato-biliary-Pancreatic Surgery, Southwest Hospital Affiliated to Third Military Medical University, Chongqing, China; 2 Dept. Ultrasound, Southwest Hospital Affiliated to Third Military Medical University, Chongqing, China; 3 Dept. Pathology, Southwest Hospital Affiliated to Third Military Medical University, Chongqing, China; Taipei Veterans General Hospital, TAIWAN

## Abstract

**Objectives:**

Hepatic angiomyolipoma (HAML) is a rare, benign mesenchymal tumor of the liver and its diagnosis has been considered challenging. The aim of this study was to investigate prospectively the diagnostic efficacy of the incorporation of both baseline ultrasound (US) and contrast-enhanced ultrasound (CEUS) features of HAML in patients without cirrhosis.

**Materials and Methods:**

Consecutive 1748 non-cirrhotic patients with focal liver lesions (FLLs) were prospectively enrolled. Baseline US and CEUS were performed before resection or biopsy. Ultrasound imaging diagnosis of FLLs was compared with the pathological results.

**Results:**

Final diagnoses were established in 41 patients with HAML (2.3%) with normal alpha fetal protein (AFP) level and in 1707 patients with FLL other than HAML. Diagnostic criteria for HAML was based on the combination of baseline US and CEUS appearance of the nodule: (1) Well-defined, marked hyper-echoic nodule without surrounding hypo-echoic halo on baseline US; (2) hyper-enhancement in the arterial phase (exclude initial peripheral nodular enhancement and spoke-wheel arteries) and remains hyper-enhancement or iso-enhancement in the late phase. The diagnostic criteria were fulfilled in 31 HAMLs, 1 hepatocellular adenoma and 1 hemangioma. Ten HAMLs were misdiagnosed as other liver tumors because they did not meet the diagnostic criteria mentioned above and consequently yielded a sensitivity, specificity, positive predictive values, negative predictive values and Youden index of 75.61%, 99.88%, 93.94%, 99.42%, and 0.75 respectively.

**Conclusion:**

The combination of baseline US and CEUS may lead to the correct diagnosis noninvasively in the majority of HAMLs in non-cirrhotic patients with normal AFP level.

## Introduction

Hepatic angiomyolipoma (HAML) is a rare, commonly considered to be benign mesenchymal tumor of the liver [1], containing variable proportions of adipose tissue, smooth muscle cells, and blood vessels [2,3]. HAML occurs usually as a solitary tumor in the non-cirrhotic liver and predominately in women [4,5]. Although most patients with HAML have benign clinical course, HAMLs should be considered to have uncertain malignant potential for some of them have been found to show malignancy-like histological features after reappraisal of morphological features and clinicopathological findings in recent years [3]. Actually, malignant HAMLs with extrahepatic metastases and recurrent cases after surgical resection have been reported in a few patients [6,7,8, 9]. Therefore, noninvasive characterization of the tumor is of clinical importance since careful follow-up of patients is recommended for HAML [3] and surgical intervention is suggested in some cases [5]. Conventional ultrasound (US) is usually the first-line imaging technique for liver because of its relatively low cost and high availability worldwide, however, the ability of US alone to diagnose HAML is disappointing [5]. Contrast-enhanced computed tomography (CECT) and magnetic resonance imaging (MRI) have been used for diagnosis of HAML and some imaging features proven to be helpful, however, correct preoperative diagnosis was usually achieved in less than 40% of the cases [5,6]. HAMLs have been detected more frequently for the increasing of imaging examination in clinical practice, but the pre-operative diagnosis of the tumor is still challenging. Contrast-enhanced ultrasound (CEUS) have been proven to significantly improve the characterization of focal liver lesions in most cases [10,11]. Some imaging features of HAML on CEUS have been described in recent years [12,13,14], and these imaging features were considered to be helpful in characterization of HAMLs. However, these reports were based on retrospective review of images of HAMLs on CEUS, and more importantly, these CEUS features have not been evaluated prospectively up to now. Therefore, the purpose of our study was to investigate prospectively the diagnostic efficacy of HAML using both baseline US and CEUS in a real clinical setting.

## Patients and Methods

The study was approved by the ethics committee of Southwest hospital. Written informed consent according to the ethical guidelines from Helsinki was obtained from all patients. The patients were recruited prospectively between January 2008 and June 2014. As part of our standard work-up, patient with incidentally detected focal liver lesion receive a baseline US and CEUS examination. The following inclusion criteria were applied:

1. Focal liver lesion detected by ultrasound in non-cirrhotic patients
2. Patient underwent CEUS for characterization of focal liver lesion before resection or biopsy
3. Pathological diagnosis of focal liver lesion was established through assessment of specimen from resection or biopsy

Exclusion c+riteria were:

1. Systemic chemotherapy or targeted therapy prior to the CEUS.
2. Pathological diagnosis of focal liver lesion was indeterminate after biopsy
3. Patients with cirrhosis because solid mass found in cirrhotic liver are highly suspicious for hepatocellular carcinoma and inconclusive CEUS patterns does not rule out malignancy in cirrhotic liver.

**Fig 1:**
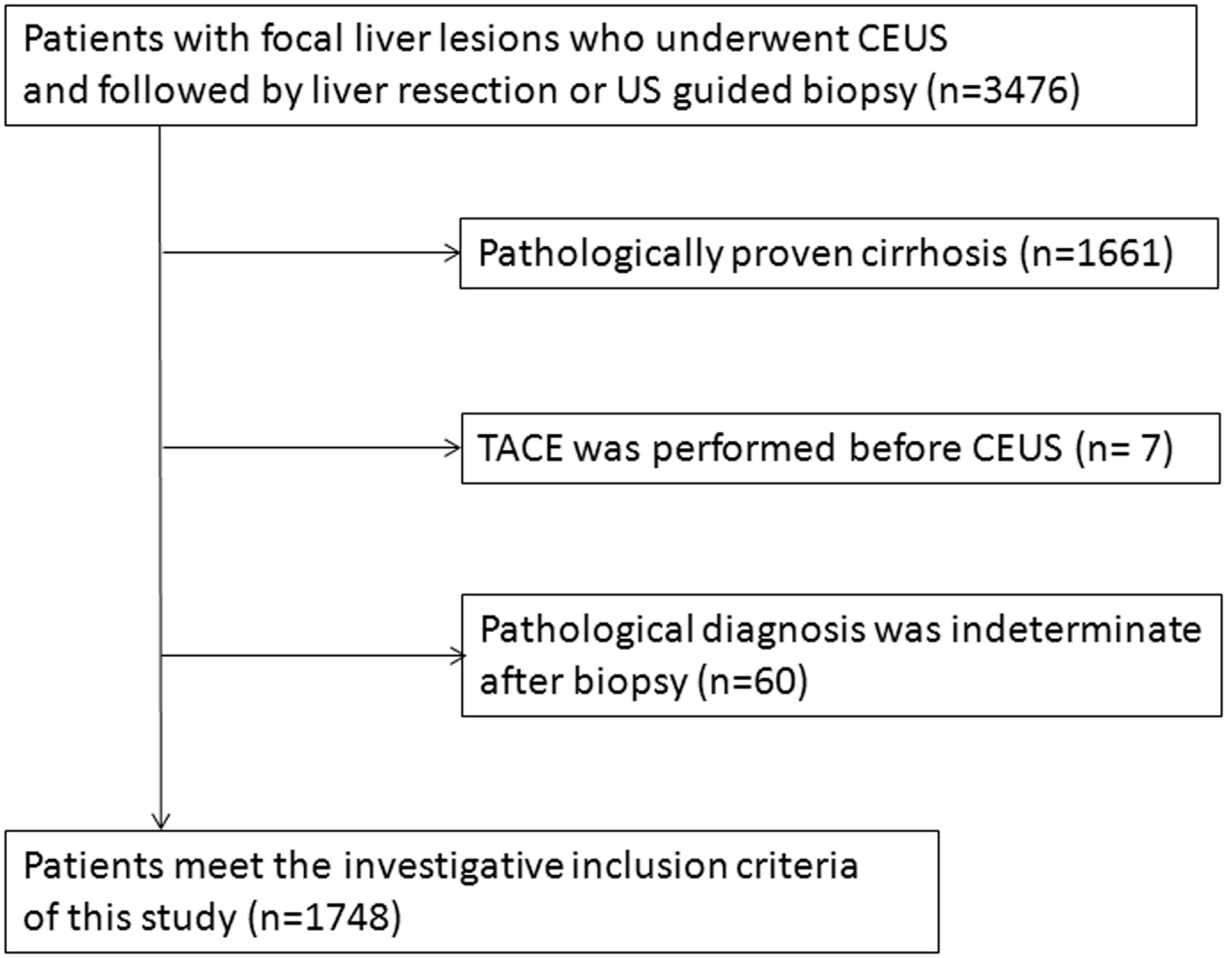
Flowchart shows inclusion and exclusion of patients for this study.

According to the inclusion and exclusion criteria of this study, 1731 patients were excluded for various reasons, and the final analysis was performed in 1748 patients (Fig 1: flowchart shows inclusion and exclusion of patients for this study).

### US examination and criteria for classification of HAML

Baseline US and CEUS were performed with a mutifrequency 4C1 convex array probe of an Acuson Sequoia 512 ultrasound unit (Simens Medical Solutions, Santa Clara, Calif) as described in detail previously [12,15]. The enhancement course of the target nodule during arterial, portal, and late phases was digitally recorded and evaluated from video clips by two experienced physicians (R L, and XH Z)who had over 15 year experience of liver US scanning and diagnosis before surgical resection or biopsy. Baseline US and CEUS images were systematically reviewed in a common reading to reach a consensual judgment when discordant diagnosis between both physicians had occurred. Ultrasound definition for HAML was based on the combination of baseline US and CEUS appearance of the nodule: (1) Well-defined, marked hyper-echoic nodule (heterogeneous or homogeneous) without surrounding hypo-echoic halo on baseline US; (2) intense hyper-enhancement in the arterial phase (exclude initial peripheral nodular enhancement and spoke-wheel arteries) and remains hyper-enhancement or iso-enhancement in the late phase [12,13]. A per-patient analysis was performed in this study. Only the largest one was taken into consideration if a patient had more than one lesion in the liver.

### CT scan

Abdomen CT was performed with 64-multidetector CT (MDCT, Definition, Siemens, Erlangen, Germany) using a 3 phase contras-enhanced protocol. First, an unenhanced scan was obtained through the liver. Next, after intravenous infusion of 100–120 ml of a nonionic iodine-containing contrast agent (ultravist 370, Scherning AG, Berlin, Germany) using a power injector (Stellant CT Injection System, Medrad, Indianola, Pennsylvania) at a rate of 4.0 ml /sec. Contrast-enhanced scans were obtained in arterial with bolus test trigger for optimal characterization of focal hepatic lesions. Data acquisitions were obtained through the whole liver in a craniocaudal direction during a single breath-hold helical acquisition for 4–6 sec with 5 mm slice thickness and 0.5 s rotation time. The acquisition of the arterial phase was automatically started 5 s after contrast agent reaching the threshold in the aorta. The start of acquisition sequences was 60 s for the portal venous phase and 180 s for the delayed phase. Densities of less than -20HU on unenhanced CT scan was used to confirm the presence of fat in the lesion [15]. We defined the enhancement patterns according to the predominant enhancing pattern occupying more than 50% of area of the lesion compared to adjacent hepatic parenchyma at contrast enhanced CT. Washout was defined as hyperdense of the lesion during the arterial phase followed by hypodense in the portal or late phase on contrast enhanced CT.

### The diagnostic algorithm of the liver nodules

The diagnostic gold standard was histology through a specimen of surgical resection or biopsy. Clinical situation of the patient was thoroughly considered and the option of the patient was fully respected in this diagnostic work up. No preoperative biopsy was performed in patients who had decided to receive surgical resection. US-guided biopsy was performed in patients who refuse liver resection without pathological diagnosis of the nodule. US-guided biopsy was performed using an18gauge needle (Bard Peripherals Vascular Inc, Tempe, Arizona 85281, U S A) within the nodule and the surrounding liver parenchyma. Specimens were routinely fixed with formalin, imbedded in paraffin and stained with hematoxylin and eosin. Liver sections were examined by pathologists with over 20 year experience of liver pathology (XCY and DYG) who were blinded to the results of the clinical and radiological examinations. Diagnosis of HAML was made when varying amounts of smooth muscle cells, adipose tissue, and blood vessels are observed in a hepatic specimen, in addition, positive for homatropine methylbromide–45with immunohistochemical stains. Final pathological diagnosis was made in consensus by experienced pathologists (XCY and DYG).

### Statistics

Baseline characteristics of the patients are expressed as median and range or count and proportion. True-positive—that is, the lesion was correctly assessed as HAML by US and CEUS according to the diagnostic criteria mentioned above before resection or biopsy; false-negative—that is, when a HAML was incorrectly assessed as non-HAML by US and CEUS; true negative—that is, a non-HAML lesion was correctly judged to be focal liver lesion other than HAML by US and CEUS; false positive—that is, a non-HAML lesion was incorrectly assessed as HAML by US and CEUS. Sensitivity, specificity, positive predictive value, negative predictive value, and Youden index were calculated. Student t test for continuous variables and Chi-Squared test for categorical variables were used for comparison. A *P* value of less than 0.05 was considered statistically significant. Statistical analysis were performed using the SPSS 13.0 software package (SPSS Inc, Chicago, IL) [16].

## Results

Between 1, January 2008 and, 30, June 2014, a total of 1748 patients with histologically diagnosed focal liver lesions were included in this cohort. One thousand and ninety three patients with focal liver lesions were diagnosed as malignant tumors (including 747 patients with hepatocellular carcinoma, 198 intrahepatic cholangiocarcinoma, 118 metastases, 9 carcinosarcoma,6 sarcoma, 4 lymphoma, 3neuroendocrine carcinoma, 2 hepatoblastoma, 3 epithelioid hemangioendothelioma, 3 other malignant tumors), whereas 655 patients were diagnosed as benign lesions (including 294 patients with hemangioma, 95 focal nodular hyperplasia, 61 pyogenic liver abscesses, 54 inflammatory pseudotumor, 41 hepatic angiomyolipoma, 23 hydated infection, 1amebic liver abscess, 17 liver cysts complicated by hemorrhage or infection, 11 hepatic adenoma, 10 hepatic tuberculosis, 10 focal fatty change, 10 dysplastic nodule, 9 solitary necrotic nodule, 8 chronic hepatitis, 5 biliary cystadenoma, 3 cholangiocellular adenoma, 1 biliary hamartoma,1 myelolipoma and 1 lymphangioma). Patients with HAML constitute 2.3% of all patients enrolled in this study. Clinical characteristics of 41 patients with histologically proven HAML are demonstrated in Table 1. The mean age of patients was 46 ±11 years. Majority of the patients (75.6%) was female (male: female = 1:3.1). Twenty five patients (60.9%) were symptomless, 13 patients complained of right upper abdominal discomfort or abdominal pain, 3 patients had abdominal fullness and palpable mass. A history of renal angiomyolipoma or tuberous sclerosis was not found in any patient. Ten patients (24.4%) had positive serum test for hepatitis B surface antigen and serum alpha-fetoprotein level was within normal range. The mean size of lesions was 6.2 ±4.4cm. Most the lesions (85.4%) were solitary and located more frequently in the right lobe of the liver (58.3%). The final diagnosis of HAMLs was confirmed in all patients after examination of surgical specimens (in 35 patients) or biopsy (in 6 patients).

**Table 1:** Demographic characteristics of 41patients with HAML in this study. HBV: hepatitis B virus; HCV: hepatitis C virus; AFP: Alpha-fetoprotein. HBV maker positive: positive serum test for hepatitis B surface antigen; HCV maker positive: positive test for antibody to hepatitis C virus; Alcohol drink: daily intake of >50 g of ethanol >5 years.

Age median[range] (years)	45 (27–70)
<30	2
31–50	26
>50	13
Gender	
Male	10
Female	31
Related history	
HBV maker positive	10
HCV maker positive	0
Oral contraceptive use	0
Glycogen storage diseases	0
Alcohol drink	6
AFP median [range] (ng/ml)	2.16 (1.04–13.44)
≤ 20 ng/ml	41
>20 ng/ml	0
Location of lesions	
Left hepatic lobe	11
Right hepatic lobe	24
Both left and right lobe	6
Number of lesions / patient	
1 lesion	35
2 lesions	2
≥3 lesions	4
Size of lesions median[range](cm)	5.2 (1.6–25)
≤30 mm	11
31–50 mm	9
> 50 mm	21

### US findings

Characteristics of HAMLs on baseline US are summarized in Table 2. Most HAMLs were markedly hyper-echoic (80.5%) and inhomogeneous (80.5%). All lesions were smooth and well-defined. No hypo-echoic halo was displayed. Punctiform and/or filiform blood flow was depicted by color Doppler in most lesions (87.8%). Real-time contrast enhancing patterns of 41 histologically proven HAMLs are demonstrated in Table 3. The dynamic enhancing patterns of the lesions throughout the different vascular phases of the study were classified into 4 types: (1) sustained enhancement pattern—33 HAMLs displayed hyperenhancement in the arterial phase and remained hyperenhancement or isoenhancement in the late phase; (2) Rim-like enhancement pattern—2 HAMLs showed peripheral rim-like hyperenhancement in the arterial phase and remained hyperenhancement in the late phase with no enhancement at internal part of the lesion during all the three phases; (3) wash-out enhancement pattern—5 HAMLs displayed hyperenhancement in the arterial phase and followed by hypoenhancement in the late phase; (4) hypoenhancement pattern—1 HAML demonstrated hypoenhancement during all the three vascular phases.

**Table 2:** Gray scale and color Doppler imaging characteristics of 41 HAMLs.

Characteristics	Number of lesions
Echogenicity	
Hyperechoic	33
Isoechoic	1
Hypoechoic	7
Inhomogeneous	33
Homogeneous	8
Tumor margin	
Smooth and well-defined	41
Hypoechoic halo	0
Ill-defined	0
Color Doppler imaging	
Peripheral blood flow	11
Internal blood flow	5
Peripheral and internal flow	20
No blood flow visualized	5

**Table 3:** Real-time contrast-enhancing pattern of 41 histologically proven HAMLs.

Enhancing pattern	Arterial phase	Portal phase	Late phase
Hyperenhancement			
Inhomogeneous	31	25	23
Homogeneous	7	3	0
Rim-like hyperenhancement	2	2	2
Isoenhancement	0	6	10
Hypoenhancement	1	5	6

**Fig 2:**
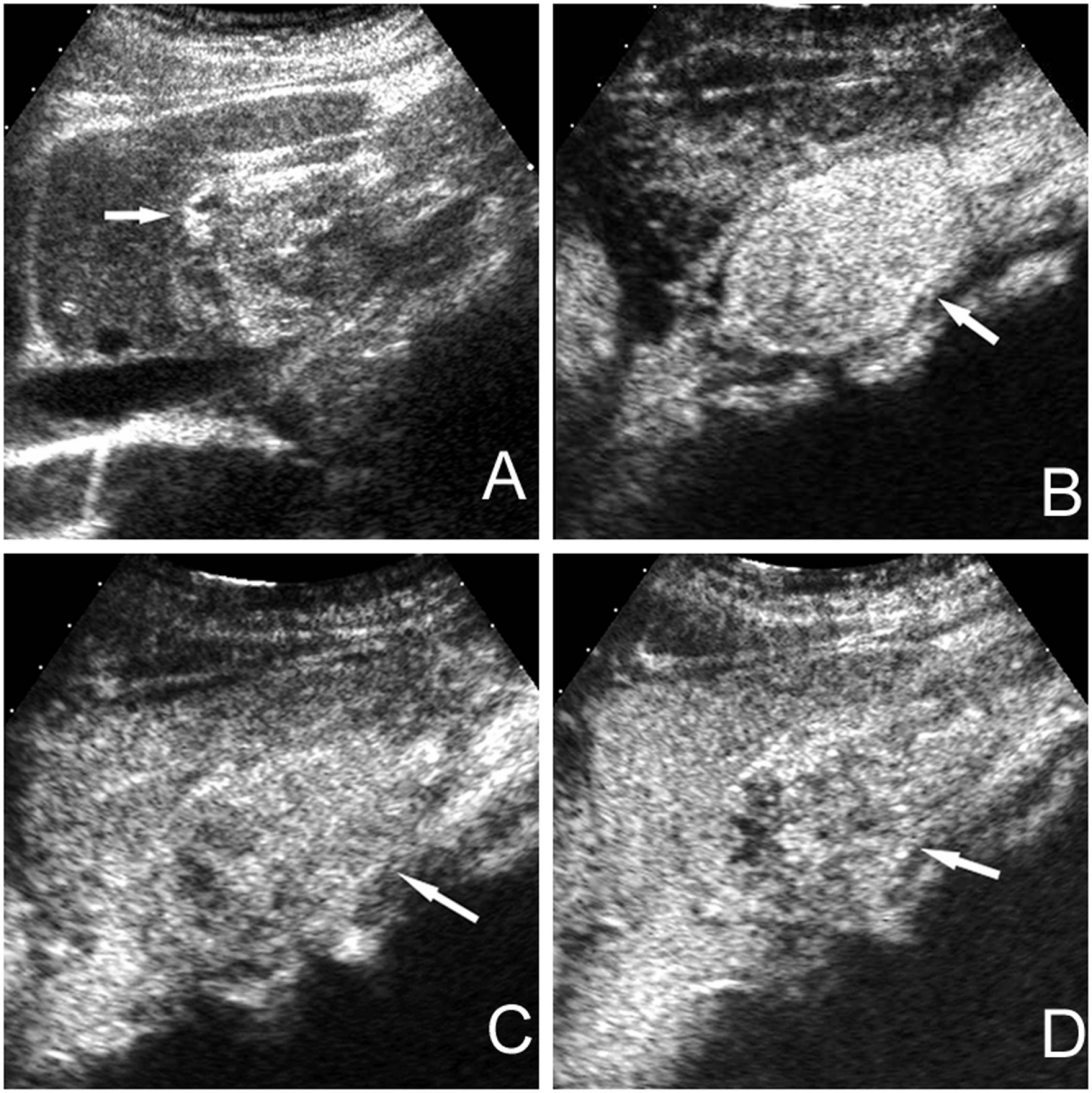
Typical appearance of HAML on baseline US and CEUS. There is a well-defined, marked heterogeneous hyper-echoic nodule of 44 mm×31mm in size without surrounding hypo-echoic halo on baseline US in the left lobe of liver (white arrow) in a 39 year female patient (A). The nodule shows heterogeneous hyper-enhancement in the arterial phase (13 seconds after injection of contrast agent) without initial peripheral nodular enhancement or spoke-wheel arteries (B). The nodule shows heterogeneous hyper-enhancement in the portal phase (1 minute and 21 seconds after injection of contrast agent) and sustained heterogeneous hyper-enhancement in the late phase. (3 minutes and 17 seconds after injection of contrast agent,) (C,D).

Thirty three well-defined lesions showing marked hyper-echogenicity on baseline US and sustained enhancement pattern without initial peripheral nodular enhancement or spoke-wheel arteries on CEUS had been diagnosed as HAML before liver resection or biopsy (Fig 2). Subsequent pathological analysis turned out to be HAML in 31 patients, hepatocellular adenoma in one patient and hemangioma in another patient. Ten HAMLs were misdiagnosed as other liver tumors because they did not fulfill the diagnostic criteria mentioned above: 5 lesions (4 hypoechoic and 1 hyperechoic on baseline US) showing wash out enhancement pattern were diagnosed as malignant tumors, 1 large cystic lesion and 1 inhomogeneous hypoechoic lesion showing rim-like enhancement pattern were interpreted as benign liver tumor with internal haemorrhage and necrosis, 1 isoechoic lesion and 1 hypoechoic lesion showing sustained enhancement pattern were characterized as benign liver tumor, and 1 marked hyperechoic lesion showing hypoenhancement during all the three vascular phases was interpreted as hypovascular tumor with indeterminate diagnosis. Other 1705 focal liver lesions (99.88%) that did not meet the diagnostic criteria of HAML were correctly interpreted as non-HAML lesions.

The sensitivity, specificity, positive predictive values, negative predictive values and Youden index for HAML diagnosis by CEUS were 75.61%, 99.88%, 93.94%, 99.42%, and 0.75 respectively.

There were no statistically significant differences between 31 patients with HAML correctly diagnosed by CEUS and 10 patients with HAML incorrectly diagnosed by CEUS in age, sex, positive serum test for hepatitis B surface antigen, serum AFP level, and tumor size (Table 4).

**Table 4:** Demographic characteristics of 31patients with HAML correctly diagnosed by CEUS and 10 patients with HAML incorrectly diagnosed by CEUS. HBV: hepatitis B virus; HBV maker positive: positive serum test for hepatitis B surface antigen; AFP: Alpha-fetoprotein.

	Correctly diagnosed(n = 31)	Incorrectly diagnosed(n = 10)	*p* value
Age(±s) (years)	45.68 ±11.49	43.20±10.49	0.545
median[range] (years)	46 (27–70)	41 (31–65)	
Gender			1.000
Male	6 (19%)	2 (20%)	
Female	25 (81%)	8 (80%)	
Related history			
HBV maker positive	5 (16%)	3 (30%)	0.615
AFP (±s) (ng/ml)	2.19±0.8	2.45±0.85	0.398
median [range] (ng/ml)	2.09 (0.24–4.21)	2.16 (1.11–4.01)	
Size of lesions (±s) (cm)	6.66±4.45	4.19± 2.59	0.105
median [range](cm)	5.7 (1.6–25)	2.75 (1.9–8)	
Location of lesions			0.956
Left hepatic lobe	8	3	
Right hepatic lobe	18	6	
Both left and right lobe	5	1	
Number of lesions / patient			0.68
1 lesion	25	10	
2 lesions	2	0	
≥3 lesions	4	0	

### CT findings

Contrast—enhanced CT was performed in 38 patients with HAML, and MRI in 1 patient. All 38 HAMLs was seen as well-defined hypodense mass in the liver on unenhanced CT and fat attenuation was revealed in 27 lesions (71.1%). Arterial hyper-enhancement was seen in 27 lesions (71.1%). Nine lesions (23.7%) displayed hypo-enhancement and 2 lesions non-enhancement (5.3%) during the arterial phase. Washout was observed in 18 lesions (47.4%) during the late phase on contrast enhanced CT. Ten HAMLs without fat attenuation and 6 HAMLs with small fat attenuation showing arterial hyper-enhancement followed by wash out in the portal or late phase were incorrectly diagnosed as hepatocellular carcinoma. One HAML without fat attenuation showing heterogeneous hyper-enhancement during the arterial, portal and late phases was incorrectly diagnosed as hemangioma. Three HAMLs with predominant fat attenuation showing non-enhancement (2 lesions) or marked hypo-enhancement during all the vascular phases were misdiagnosed as lipoma. One HAML without fat attenuation and 1 HAML with very small fat attenuation showing arterial hyper-enhancement and iso-enhancement in the late phase were judged as indeterminacy. Five HAMLs with fat attenuation showing heterogeneous hyper-enhancement during all the three vascular phases were correctly diagnosed as HAML. Nine HAMLs with fat attenuation showing hypo-enhancement during all the three vascular phases were correctly diagnosed as HAML. Two HAMLs with fat component occupying about 60%-70% of the lesion showing arterial hyper-enhancement followed by wash out in the portal phase were correctly judged as HAML. Contrast—enhanced CT was performed in 1465 patients out of 1704 patients with focal liver lesion other than HAML enrolled in this study. Six patients with focal liver lesion other than HAML (2 well-differentiated HCC, 1 liposarcoma, 1 hepatic adenoma, 1 focal nodular hyperplasia and 1hemamgioma) were misdiagnosed as HAML because a significant amount of fat component was detected on CT. One thousand four hundred and fifty nine patients with focal liver lesions other than were correctly interpreted as non-HAML lesions after CT scanning. The sensitivity, specificity, positive predictive values, negative predictive values and Youden index for HAML diagnosis by CT were 42.11%, 99.59%, 72.73%, 98.51%, and 0.42 respectively.

When compared with CEUS, the sensitivity (*p* = 0.002) and negative predictive values (*p* = 0.011) of CT were significantly lower than that of CEUS, however, the specificity (*p* = 0.155) and the positive predictive values (*p* = 0.049) of CT were similar to that of CEUS.

Thirty eight patients with HAML received both CEUS and CT examinations before resection or biopsy. The paired diagnostic outcomes of the 38 patients are shown in Table 5. Concordance between CEUS and CT was observed in 18/38 cases (47.4%).

**Table 5:** Paired contingency of CEUS and CT in the diagnosis of HAML in 38 patients.

	CT Correct	CT Incorrect
CEUS correct	12	16
CEUS incorrect	4	6

## Discussion

Most of HAMLs (80.5%) in this study demonstrated marked hyper-echogenicity, dominantly inhomogeneous on baseline US. These echogenic features were in accordance with previous reports [12,13,14] and may be explained by the special histological composition of HAML. HAMLs are composed of variable amount of fat, blood vessels and smooth muscle tissues and the three types of tissues are heterogeneously mixed in the lesion. With all the three tissues interlace each other, multi-interface echo can be displayed by ultrasound, which may result in heterogeneous higher echogenicity compared to surrounding hepatic parenchyma on baseline US [12]. In the present study, the majority of HAMLs (80.5%) revealed sustained enhancement pattern on CEUS, namely hyperenhancement in the arterial phase and remained hyperenhancement or isoenhancement in the late phase and this enhancement pattern has also been documented in previous retrospective observations of pathologically proven HAMLs [12,13,14]. Sustained enhancement has been considered as typical pattern in solid benign liver tumors [17,18,19] and the diagnosis of malignant liver tumor has been averted practically in those HAMLs showing sustained enhancement on CEUS in our series. The combination of the imaging features of both baseline US and CEUS results incorrect preoperative diagnosis in the majority (75.6%) of HAMLs in this prospective cohort under a real clinical setting. Nevertheless, some cases of HAMLs were misdiagnosed as other FLLs for they do not meet our diagnostic criteria of HAML. These lesions lead to 24.4% of false negative diagnosis of HAML. Therefore, biopsy is still necessary to establish a definite diagnosis if typical characteristics of HAML not displayed on baseline US and CEUS. In those lesions showing both marked hyper-echogenicity on baseline US and sustained enhancement on CEUS, pathological analysis turned out hepatocellular adenoma in one patient and hemangioma in another patient, yielding only 4.9% of false positive diagnosis which is acceptably low in real clinical practice and follow up for those patients is recommended.

The typical CEUS features of a hemangioma are peripheral nodular enhancement in arterial phase, followed by centripetal enhancement to partial or complete fill-in of the lesion, which allow an accurate diagnosis of hemamgioma in 95%-98% of cases [10,20]. Peripheral nodular enhancement in the arterial phase was excluded from the diagnostic criteria of HAML in this study, therefore, most hemangiomas have been correctly distinguished from HAMLs. Although in a few cases of so called “shunt-hemangiomas” (also described as high-flow hemangiomas) may display homogenous arterial enhancement [20], most of the shunt-hemangiomas are hypo-echoic on baseline US and arterial enhancement usually initiate at the periphery with subsequent very rapid centripetal filling when early arterial enhancement course are attentively reviewed which is obviously different from that of HAML on real time CEUS. Actually, only one case of hemangioma (0.3%) showing both marked hyper-echogenicity on baseline US and sustained enhancement on CEUS was misdiagnosed as HAML in this large series.

Focal nodular hyperplasia (FNH) typically displays as hyperenhancing lesions in all phases. Centrifugal filling or spoke-wheel arteries could be detected in about 70% of all cases [21,22]. More importantly, most FNHs appeared isoechoic or hypoechoic on baseline US which is quite different from that of typical HAMLs. Therefore, none of the 95cases of FNH was incorrectly judged as HAML in the present study.

Hepatocellular adenoma is a rare benign liver tumor and predominantly diagnosed in young women with a prolonged history of oral contraceptive use [23]. None of 41 cases of HAMLs had a long history of estrogen based, oral contraceptive steroid use in our series, which may be attributed to lesser use of oral contraceptives in China than in Europe and North America [24] and may add useful information in differentiating HAML from hepatocellular adenoma. Contrary to typical HAML, hepatocellular adenomas appear predominantly hypoechoic or isoechoic on baseline US, and show rapid hyperenhancement in the arterial phase following a decent wash out in the late phase due to missing portal veins of the tumor [21]. In fact, only one case of hepatocellular adenoma showing both marked hyper-echogenicity on baseline US and sustained enhancement on CEUS which was incorrectly interpreted as HAML in our study.

Focal fatty infiltration shows marked hyper-echogenicity with well-defined margin may mimic neoplastic lesion. However, focal fatty infiltration commonly locates at the periportal region of liver and lack of mass effect, more importantly, focal fatty infiltration displays exactly the same enhancement patterns as the adjacent liver parenchyma in all the vascular phases [19, 25], therefore, could be distinguished confidently from HAML by CEUS.

In the present study, the diagnosis of CEUS was not in agreement with that of CT in over half cases of HAML (52.6%). This discrepancy may be attributed mainly to the difference in pharmacokinetics of ultrasound contrast agents in comparison to those for CT [26] and consequently discordant enhancing behavior between CEUS and CT in focal liver lesions. In our study, washout (hyperenhancement in the arterial phase and followed by hypoenhancement in the late phase) was observed in much more cases of HAML at CT (18/38, 47.4%) than on CEUS (5/38, 13.2%) which is liable to incorrect diagnosis of malignant tumor. The secondary reason for the discrepancy may be the detection of fat component at CT which is helpful in the diagnosis of HAML though approximately one-third of HAML contained fat in less than 10% of their area [27]. In the present study, 4 cases of HAML (10.5%) not showing typical features on CEUS was correctly judged as HAML by CT because apparent fat component was observed at CT. In our opinion, in case CEUS and CT findings were not consistent and alpha fetal protein normal in patient without cirrhosis, US guided biopsy of liver tumor is still recommended to establish the diagnosis.

We acknowledge that some limitations exist in this study. First, the limited sample size reflects the low incidence of HAML, though the current number of HAMLs was the result of a cohort of 1748 histologically diagnosed focal liver lesions in non-cirrhotic patients who attended our referral center. Second, this work is single center based investigation and ultrasound appearances of focal liver lesions were judged by two experienced doctors, therefore multicenter study with more patients is needed to verify the results of our study in the future work.

In summary, typical HAML displayed as marked hyper-echoic tumor with smooth and well-defined margin without surrounding hypo-echoic halo on baseline US, hyperenhancing in arterial phase and remained hyperenhancement or isoenhancement in the late phase on CEUS. The combination of baseline US and CEUS may lead to the correct preoperative diagnosis noninvasively in the majority of HAMLs in non-cirrhotic patients with normal alpha fetal protein level.

## Supporting Information

S1 STARD Checklist (PDF)
