# Supplementary material for: Diagnosis of Hepatic Angiomyolipoma by Combination of Baseline and Contrast-Enhanced Ultrasound—A Prospective Study in Non-Cirrhotic Patients
Source: PLoS One. 2015 Jul 6;10(7):e0132290. doi: 10.1371/journal.pone.0132290 (PMC4492985; doi:10.1371/journal.pone.0132290)
Supplement: S1 STARD Checklist — (PDF) [file pone.0132290.s001.pdf]

STARD checklist for the reporting of studies of diagnostic accuracy.

First official version, January 2003.

Section and Topic	Item #		On page #
TITLE/ABSTRACT/ KEYWORDS	1	Identify the article as a study of diagnostic accuracy (recommend MeSH heading 'sensitivity and specificity').	1, 2
INTRODUCTION	2	State the research questions or study aims, such as estimating diagnostic accuracy or comparing accuracy between tests or across participant groups.	4
METHODS			
Participants	3	Describe the study population: The inclusion and exclusion criteria, setting and locations where the data were collected.	4, 5
	4	Describe participant recruitment: Was recruitment based on presenting symptoms, results from previous tests, or the fact that the participants had received the index tests or the reference standard?	4
	5	Describe participant sampling: Was the study population a consecutive series of participants defined by the selection criteria in items 3 and 4? If not, specify how participants were further selected.	4
	6	Describe data collection: Was data collection planned before the index test and reference standard were performed (prospective study) or after (retrospective study)?	4
Test methods	7	Describe the reference standard and its rationale.	5
	8	Describe technical specifications of material and methods involved including how and when measurements were taken, and/or cite references for index tests and reference standard.	5
	9	Describe definition of and rationale for the units, cutoffs and/or categories of the results of the index tests and the reference standard.	5
	10	Describe the number, training and expertise of the persons executing and reading the index tests and the reference standard.	5
	11	Describe whether or not the readers of the index tests and reference standard were blind (masked) to the results of the other test and describe any other clinical information available to the readers.	5, 6
Statistical methods	12	Describe methods for calculating or comparing measures of diagnostic accuracy, and the statistical methods used to quantify uncertainty (e.g. 95% confidence intervals).	6
	13	Describe methods for calculating test reproducibility, if done.	
RESULTS			
Participants	14	Report when study was done, including beginning and ending dates of recruitment.	7
	15	Report clinical and demographic characteristics of the study population (e.g. age, sex, spectrum of presenting symptoms, comorbidity, current treatments, recruitment centers).	7
	16	Report the number of participants satisfying the criteria for inclusion that did or did not undergo the index tests and/or the reference standard; describe why participants failed to receive either test (a flow diagram is strongly recommended).	7
Test results	17	Report time interval from the index tests to the reference standard, and any treatment administered between.	7
	18	Report distribution of severity of disease (define criteria) in those with the target condition; other diagnoses in participants without the target condition.	7
	19	Report a cross tabulation of the results of the index tests (including indeterminate and missing results) by the results of the reference standard; for continuous results, the distribution of the test results by the results of the reference standard.	8
	20	Report any adverse events from performing the index tests or the reference standard.	
Estimates	21	Report estimates of diagnostic accuracy and measures of statistical uncertainty (e.g. 95% confidence intervals).	9
	22	Report how indeterminate results, missing responses and outliers of the index tests were handled.	8
	23	Report estimates of variability of diagnostic accuracy between subgroups of participants, readers or centers, if done.	
	24	Report estimates of test reproducibility, if done.	
DISCUSSION	25	Discuss the clinical applicability of the study findings.	12
